# Evolution of COVID-19 Pregnancies Treated With Nitazoxanide in a Third-Level Hospital

**DOI:** 10.7759/cureus.15002

**Published:** 2021-05-13

**Authors:** Krista Yazareth Enríquez López, José Meneses Calderón, Lilia de la Cruz Ávila, Miguel Ángel López Esquivel, Jazmín Meneses Figueroa, María José Vargas Contreras, José Anaya Herrera, Ofelia Roxana Sotelo Martínez, José Antonio Mendoza López, Hugo Mendieta Zerón

**Affiliations:** 1 Department of Obstetrics and Gynaecology, “Mónica Pretelini Sáenz” Maternal-Perinatal Hospital, Toluca, MEX; 2 Department of Research, “Mónica Pretelini Sáenz” Maternal-Perinatal Hospital, Toluca, MEX; 3 Department of COVID, “Mónica Pretelini Sáenz” Maternal-Perinatal Hospital, Toluca, MEX; 4 Department of Telemedicine, “Mónica Pretelini Sáenz” Maternal-Perinatal Hospital, Toluca, MEX; 5 Laboratory of Genetics, Faculty of Medicine, Autonomous University of the State of Mexico, Toluca, MEX; 6 Research and Teaching Unit, “Mónica Pretelini Sáenz” Maternal-Perinatal Hospital, Toluca, MEX; 7 Reseach and Teaching Unit, “Mónica Pretelini Sáenz” Maternal-Perinatal Hospital, Toluca, MEX; 8 Faculty of Medicine, Autonomous University of the State of Mexico, Toluca, MEX

**Keywords:** nitazoxanide, covid-19, pregnancy, puerperium

## Abstract

Background

Nitazoxanide shows adequate *in vitro* activity against coronavirus. The aim of this study was to describe the behavior of coronavirus disease 2019 (COVID-19) in pregnant women treated with nitazoxanide.

Methodology

This cross-sectional study included the files of COVID-19 positive pregnant women treated with nitazoxanide 500 mg every 6 hours, levofloxacin every 12 hours, and clarithromycin 500 mg every 12 hours.

Results

The data of 51 women (mean age: 27.4 ± 7.2 years) were analyzed. Eleven (21.56%) patients had to receive medical attention in the intensive care unit. There were 22 (43.13%) preterm deliveries, 21 by cesarean and one by vaginal delivery. The medical attention of this population was as follows: 31 cesareans, five vaginal deliveries, nine still pregnant, two requiring manual vacuum aspiration, two ectopic pregnancies, one requiring curettage, and one requiring hysterotomy. There were seven (13.72%) cases of preeclampsia, and there were two (3.92%) deaths.

Conclusion

Nitazoxanide prescription could be an option against COVID-19 in pregnancy due to its safety profile.

## Introduction

Pregnancy causes physiological and immunological changes that make women more susceptible to infectious processes, this is especially important in viral diseases that affect the respiratory tract, predisposing women in the gestational state to unfavorable results compared to the rest of the adult population. This increase in susceptibility is believed to be a combination of adaptive changes in immunity to allow fetal allograft associated with anatomophysiological changes that accompany pregnancy [1].

Due to the recent discovery of the severe acute respiratory syndrome coronavirus 2 (SARS-Cov-2), there are limited reports of experience in pregnant women. As of this time, there are no data to report that pregnancy increases susceptibility to coronavirus disease 2019 (COVID-19), but hypothetically it is very plausible due to the change in T-helper 2 (Th2) system dominance [2].

Historically, viral pandemics have had a high mortality among pregnant women. Briefly, during the influenza pandemics of 1918 which has been considered the most devastating in the history of mankind, the estimated deaths were about 50 million [3], with a mortality of 27% in a casuistry among 1,350 pregnant women, with most of them being from the state of Maryland, USA [4]. Moreover, in a smaller series of 80 cases that occurred in Chicago, USA, the mortality was 45% [5].

During the 1957 pandemic, the mortality rate was close to 20%, astonishingly 50% of the women who died in reproductive age were pregnant. Furthermore, 10% of the deaths reported in this pandemic occurred in pregnant women, with the majority in the advanced stages of pregnancy. In both pandemics (1918 and 1957), 50% of the women who contracted the disease developed pneumonia, with mortality in this group being 50%, and in half of these cases the pregnancy was interrupted by spontaneous abortion or premature delivery with a high incidence of fetal alterations [6,7].

In the 2009 pandemic, a study in Australia documented a preterm birth rate of around 40%, especially in patients with pneumonia [8]. A fact that proves the high susceptibility of the pregnant population is that they have a greater risk of suffering complications, particularly during the second and third trimesters, in addition to the risk of hospitalization in the season of influenza, which is more frequent compared to non-pregnant or postpartum women; the risk is 3.4 to 5.1 higher, especially in the presence of comorbidities [7,9].

The immediate antecedents to the pandemic that we are now dealing with were the SARS in 2002 and the Middle East Respiratory Syndrome (MERS) caused by β-coronaviruses, which also cause serious complications in pregnancy such as abortion, premature delivery, intrauterine growth restriction (IUGR), and maternal death [10,11]. A third β-coronavirus appears in less than 20 years and is detected and notified by the Chinese government at the end of 2019, and since then it has become the worst pandemic in the last 100 years that has spread to practically all the nations of the world, affecting more than 20 million inhabitants up to the time of writing this article, with a global mortality of 737,417 people and a fatality rate of 3.7% [12].

In Mexico, since the notification of the first two cases on February 28, 2020, 156 days later there were 498,380 confirmed cases with 54,666 deaths, with a case fatality rate of 10.9% [13], placing us in the third world place of deaths only behind the United States (163,613) and Brazil (101,752) and in fourth place of fatality after the United Kingdom (14.8%), Italy (14.0%), and France (12.6%) [14].

Currently, we do not have any treatment against COVID-19 that has been shown to be significantly superior for its management, and the provision of the vaccine seems distant. This reveals that the multiple studies published have focused on patients in intermediate or advanced stages of the pathogenesis, and the effect of prevention with the 'lock-down', social distancing, and masks is complex to analyze. Based on this reasoning, repositioning nitazoxanide for its early use based on previous research that shows adequate in vitro activity against numerous viruses including coronaviruses seems reasonable.

The facts that support its use in the actual pandemic are the following scientific evidence: 1) it inhibits the viral replication of the coronavirus [15], (2) it amplifies the antiviral response of the host by enhancing the pathways of type 1 interferons (IFN-1) when exposed to exogenous cytoplasmic ribonucleic acid [16], (3) it suppresses the production of pro-inflammatory cytokines in peripheral blood mononuclear cells and inhibits the synthesis of interleukin 6 [15], (4) it has a broad biosafety and tolerability profile based on 75 million prescriptions in pediatric and adult patients with respiratory and gastroenteric viral infections and management of hepatitis B and C virus infections [17], and (5) unlike ivermectin, a fashionable drug against COVID-19, it can be used in children older than one year and in pregnant women. An excellent review of this drug has recently been published [18], and there are some promising results in the clinical field [19,20].

The nitazoxanide anti-coronavirus action is explained by the pro-inflammatory cytokines production inhibition accompanied by the viral N protein expression reduction [21]. Other mechanisms of action against SARS-CoV-2 have been described blocking the non-endosomal entry that involves the proteolytic cleavage of SARS-CoV-2 spike proteins (S1/S2) by host cell proteases transmembrane protease serine 2 (TMPRSS2) and furin, and the endosomal entry involving the recognition and binding of the receptor-binding domain (RBD) of SARS-CoV-2 spike proteins to the host cell receptors ACE2 (angiotensin-converting enzyme 2) and CD147 [22].

A recently published paper using a whole‐body physiologically based pharmacokinetic modelling to predict doses expected to maintain tizoxanide (the active metabolite of nitazoxanide) plasma and lung concentrations above the 90% effective concentration (EC90) in >90% of the simulated population reached the conclusion that optimal doses for SARS‐CoV‐2 in the fasted state were predicted to be 1,200 mg four times a day (QID), 1,600 mg three times a day (TID) and 2,900 mg two times a day (BID), and that in the fed state were 700 mg QID, 900 mg TID, and 1,400 mg BID [23].

Most of the available literature refers to previous SARS-CoV and MERS-CoV infections, inferring a possible increased risk in the mother for the development of severe disease and adverse neonatal outcomes based on these experiences. Regarding the SARS-Cov2 infection itself, the maternal clinical characteristics and symptoms of positive patients are reported emphatically, which apparently does not differ from non-pregnant patients, and it is known that the clinical characteristics show a wide range of presentation. Another main concern of the reports involves the evidence of vertical transmission of COVID-19 from the mother to the fetus, which so far has not been clear.

Apparently, the perinatal results and the clinical presentation of the COVID-19 evolution do not differ with respect to non-pregnant patients. The available information is mostly from case reports with a limited number of patients; however, it is important to add the experience of the course of pregnancy concomitant with COVID-19 in different geographic areas and different hospital settings. Alarmingly, in Mexico, the percentage of mortal cases from COVID-19 in pregnant women is around 20%. The aim of this study was to describe the behavior of COVID-19 positive pregnant women treated with nitazoxanide.

## Materials and methods

Study design

This cross-sectional study was performed capturing the information of COVID-19 positive pregnant women who attended at the “Mónica Pretelini Sáenz” Maternal Perinatal Hospital (HMPMPS), Health Institute of the State of Mexico (ISEM), Toluca, Mexico, from March to July 2020. The HMPMPS is a tertiary care referral center created with the mission of attending high-risk pregnancies. This hospital generally receives patients in the second and third trimesters of pregnancy of low socioeconomic status, without social security, and from the State of Mexico (17 million inhabitants) and the neighboring states of Guerrero and Michoacán, as well as migrants that have increased in the last five years. There are approximately 8,500 deliveries per year.

Patients

When the pandemic was confirmed by the WHO, with imminent focus on the Mexican population, the medical staff defined a general treatment as the standard of care for all pregnant (after the first trimester of pregnancy) or puerperal women based on Daxon (nitazoxanide) 500 mg every six hours, levofloxacin 500 mg every 24 hours, clarithromycin 500 mg every 24 hours, paracetamol 500 mg every eight hours in case of fever, and, if not contraindicated, enoxaparin adjusted to the patient’s weight.

By reviewing the clinical files, the inclusion criteria to follow were pregnant women or those in the puerperium infected with SARS-Cov-2 with a positive qPCR (quantitative polymerase chain reaction) test. Patients with mental compromise were excluded and incomplete medical files were discarded from the final analysis. A possible bias of the population exists because the HMPMPS is a third-level hospital with specialization in risky pregnancies.

A database on the Excel Google drive platform was created with the information collected. Quantitative variables were reported in mean ± standard deviation (SD), and qualitative variables were reported in percentages.

Ethics approval

This study was approved by the Research Ethics Committee of the HMPMPS, with current registration with the National Bioethics Commission (CONBIOETICA). The research was carried out under the ethical considerations recognized by the Declaration of Helsinki (Fortaleza, Brazil).

## Results

The files of 51 women (mean age: 27.4 ± 7.2 years; mean gestational age: 33.2 ± 7.3 weeks) were integrated and analyzed in this initial report. The general characteristics of the selected population is shown in Table 1. Eleven (21.56%) patients had to receive medical attention in the intensive care unit (ICU), and the average stay in that service was 11.88 days. There were 22 (43.13%) preterm deliveries, 21 by cesarean and one by vaginal delivery.

**Table 1:** General characteristics of the three groups BMI, body mass index; MBP, mean blood pressure

Variable	Pregnant women (N = 20)
BMI (kg/m^2^)	29.4 ± 4.9
Pregnancies (frequency)	2.1 ± 1.4
Vaginal deliveries (frequency)	0.8 ± 1.4
Abortions (frequency)	0.2 ± 0.4
Cesareans (frequency)	0.7 ± 0.8
Dyspnea (%)	66.7
Fever (%)	43.1
Headache (%)	39.2
Cough (%)	43.1
MBP (mmHg)	91.3 ± 13
Heart rate (bpm)	97.7 ± 21.1
Respiratory rate (breaths per minute)	22.5 ± 2.6
Temperature (°C)	37 ± 1.1
O_2_ saturation (%)	91.1 ± 6.8

The medical attention of this population was as follows: 31 (60.78%) cesareans, five (9.8%) vaginal deliveries, nine (17.64%) still pregnant, two (3.92%) requiring manual vacuum aspiration, two (3.92%) ectopic pregnancies, one (1.96%) requiring curettage, and one (1.96%) requiring hysterotomy. Total cases of preeclampsia were seven (13.72%).

A total of 33 live births were registered, including one case of twins, but the total number of perinatal deaths were six (perinatal mortality of 14.28% excluding those women still pregnant), two in the group of live stillbirths (one because of prematurity complications and another due to fetal bradycardia). The other cases were a baby born to a woman that was referred to the HMPMPS with the diagnosis of postpartum puerperium + hysterectomy for uterine rupture, systemic arterial hypertension, type 2 diabetes mellitus (T2DM), obesity and anemia; one in immediate puerperium born to a mother with HELLP (hemolysis, elevated liver enzymes, low platelet count) syndrome; one born to a mother who suffered from preeclampsia/eclampsia; and one case of preeclampsia with severity data and the baby with IUGR.

The main categories of patients were as follows: 18 were still pregnant and hospitalized for immediate obstetric resolution (11 otherwise healthy besides COVID-19, one with systemic hypertension, one with obesity, one with T2DM and systemic hypertension, one with lymphoma, one with hypothyroidism and IUGR, one with IUGR and oligohydramnios, and one with oligohydramnios), nine arrived and were discharged still pregnant (six otherwise healthy besides COVID-19, one with pyelonephritis, one with dengue, and one with hypothyroidism), four pregnancies were interrupted because of preeclampsia, three patients were in puerperium without preeclampsia (one with obesity, one with systemic hypertension, and one with HELLP syndrome), three patients were with incomplete abortion of the first trimester, two patients were with cervicovaginitis in pregnancy, two patients were referred to our hospital after developing preeclampsia/eclampsia in puerperium, two preterm deliveries, and a patient with one of the following conditions: (a) obstetric hemorrhage, (b) ectopic pregnancy + obstetric hemorrhage, (c) threatened preterm delivery, (d) hysterotomy, (e) puerperal woman + uterine rupture + systemic hypertension + T2DM + obesity, and (f) pregnancy interruption because preeclampsia + obstetric hemorrhage. There were two (3.92%) deaths, one arrived in immediate postcesarean puerperium, who attended a private hospital and then referred to our unit due to respiratory complications, and one last case who was still pregnant had to be interrupted because of respiratory failure from which the patient did not recover.

## Discussion

Trying to establish analogies with previous pandemics, one can remember the 2009 pandemic associated with the influenza A H1N1 virus that had its origin in a genomic mutation of the swine influenza virus. That pandemic started in April 2009 in two pediatric patients from California, USA, and almost simultaneously in a male patient in the community of La Gloria (Veracruz, Mexico), a site of great pig activity. Several studies carried out in animals showed that this new mutation had higher morbidity and mortality than the seasonal influenza virus [24], activating mechanisms indicated by the WHO, which in our country had a rapid response through the National Plan of Preparation and Response to an Influenza Pandemic [25].

There are significant differences in many aspects with the influenza outbreak of 2009, but it is surely important that at that time the correct mitigation and containment measures were taken, there was availability of a drug such as oseltamivir that was very effective for management in early stages to avoid the evolutionary process of the disease, and there was vaccine eight months after the start of the pandemic. The pandemic of 2009 caused 60.8 million cases worldwide with 18,337 deaths. In Mexico, 70,715 people, were affected with a total of 1,172 confirmed deaths. On August 18, 2011, the WHO announced the end of the pandemic, and the vaccine became available in November 2009 and its use became widespread as of 2010 [26].

In relation to the ongoing pandemic, among the first studies in pregnancy, Chen et al. [27] reported nine patients diagnosed with COVID-19 in the third trimester of pregnancy. Five (55.55%) of these patients presented with lymphopenia and all of them presented with pneumonia, although none of them required mechanical ventilatory assistance and did not report any deaths. The resolution of the pregnancy in all these patients was through cesarean section, and all the newborns had a favorable Apgar score at one and five minutes. Furthermore, vertical transmission was discarded after performing PCR tests in amniotic fluid, cord blood, pharyngeal swab, and breast milk. By comparison, in our casuistry, 36 (70.58%) of the patients had lymphopenia when arriving at our unit, and among those cases of pregnancy interruption (36 women), the percentage of cesareans was 86.11% and that of vaginal deliveries was of 13.88%.

Zhu et al. [28] reported that in nine mothers who carried COVID-19, the main symptoms were fever and cough, whereas in our study in our survey it was dyspnea (66.7%) followed by the first two with a 43.1% in both cases. They also reported that intrauterine fetal distress was observed in six (66.66%) patients, which is relatively high, as was the percentage of preterm newborns (66.66%).

Di Mascio et al. [29] carried out a systematic review that included 79 women, of whom 41 (51.9%) were infected with COVID-19, 26 (32.9%) with SARS, and 12 (15.2%) with MERS. The most common reported symptoms were fever (82.6%), cough (57.1%), and dyspnea (27.0%). In this case, the percentage of preterm deliveries (41%) was very similar to that in our data (43.13%). It is worth noting that the rate of preeclampsia was almost identical between Di Mascio et al.’s report (13.6%) and ours (13.72%). Contrary to this, the perinatal mortality in our study (14.28%) was almost double than that reported by Di Mascio et al. (7%).

Schwartz [30] after analyzing the perinatal results in 38 COVID-19 pregnant women with zero maternal deaths postulated that pregnant women were not at an increased risk of developing serious illness due to the actual pandemic. This statement is still under intense debate and scrutiny.

Zaigham and Andersson [31] carried out a systematic review of 18 articles including information of 108 pregnant patients from China, Honduras, Korea, Sweden, and the United States. The average maternal age in that study was between 29 and 32 years, mainly in the third trimester. The commonly presented symptoms were fever (68%), persistent dry cough (34%), dyspnea (12%), and diarrhea (6%), and 92% of the pregnancies ended through abdominal resolution, with the most frequent indication being fetal distress. Of the biochemical findings, the most relevant was lymphocytopenia in 59%. By contrast, we had a wider range of age, between 16 and 41 years, and the indication of cesarean due to fetal distress was low.

Another outstanding report was by Breslin et al. [32] with 43 COVID-19 positive pregnant cases, of which two cases required management in the ICU, both with obesity and comorbidities, of which one suffered from obstetric hemorrhage and the other experienced chronic arterial hypertension worsening, and both patients presented fever, with one of them requiring mechanical ventilator assistance reaching successful weaning. The difference with our approach in the number of patients requiring medical attention in the ICU (21.56%) is important and deserves more analysis, but as it is well known, the greater the variability in the studied population, the most diverse the clinical conditions and evolution one can find.

In Mexico, until epidemiological week 40 (October 5, 2020), the calculated maternal mortality ratio is 44.7 deaths per 100,000 estimated births, which represents a 32.3% increase in the ratio compared to the same week epidemiological from the previous year. From a total number of 689 accumulated maternal deaths until the first week of October 2020, the main causes of death have been confirmed COVID-19 (149 [21.6%], obstetric hemorrhage (16.7%), and hypertensive disease (16.5%), but in fourth place there were 39 (5.9%) more cases with a probable diagnosis of COVID-19. The Mexican regions with the most maternal deaths are the State of Mexico (90), Chiapas (48), Jalisco (39), Puebla (35), and Michoacán (34) [33]. According to this, maternal mortality increased around 32.3% in relation to the same epidemiological week of the year 2019, but not in the HMPMPS, where the standard treatment including nitazoxanide was established.

After more than a year since the beginning of the pandemic, most of the efforts to treat pregnant women with COVID-19 have focused on invasive ventilation support [34]. Taken in the context of other work, using remdesivir in pregnancy leads to 29% of adverse events (anemia, constipation, dysphagia, worsening hypoxia, deep vein thrombosis, etc.). Even more, in this same study by Hapshy et al., seven pregnant women abandoned the study due to increased liver function tests and serum creatinine levels [35].

The double antibiotic regimen used was based on previous data on antimicrobial sensitivity in our hospital, and it would be difficult to attribute exactly the weight in the favorable evolution of the patients. What can be mentioned as a whole is that apparently the chosen strategy has been the correct one, as in light of the current information as of May 5, 2021, with devastating numbers of maternal mortality in Mexico [36], in the HMPMPS the mortality maternal disease in 2021 is zero cases and the cumulative total since the beginning of the pandemic is 3.1%.

Despite the fact that several active vaccines are in widespread use, to get the full coverage of the worldwide population will take many years, and it must be a continuous public health policy because the SARS-CoV-2 will stay forever and having a therapeutic alternative in pregnant women, such as nitazoxanide, would be a valid option to take into account.

A clear limitation of this study is the descriptive design. Besides, differences in mean age and anthropometrics of our patients could limit the generalization of these results; notwithstanding, the evolution in this transversal study sheds light on a new alternative to treat pregnant women with COVID-19 based on the maternal and perinatal outcomes.

## Conclusions

Nitazoxanide is a cheap and widely used drug around the world that could be added to any scheme against COVID-19 in pregnancy due to its safety profile. Contrasting official numbers of maternal mortality with those retrieved from the group of patients treated at the HMPMPS, there is an astonishing difference of survival in benefit of those receiving nitazoxanide, leading to successful pregnancies with healthy babies.

Finally, as the vaccination process will still take time to include the youngest patients of childbearing age and it will also be a process that must be repeated continuously, we believe that including nitazoxanide in a management scheme for pregnant patients with COVID-19 can save thousands of lives.
